# Association between Serum Trace Elements Levels, Steroid Concentrations, and Reproductive Disorders in Ewes and Does

**DOI:** 10.1155/2022/8525089

**Published:** 2022-03-16

**Authors:** Derar Derar, Ahmed Ali, Tariq Almundarij, Essam Abd-Elmoniem, Tamim Alhassun, Moustafa Zeitoun

**Affiliations:** ^1^Department of Veterinary Medicine, College of Agriculture and Veterinary Medicine, Qassim University, Buraydah, Saudi Arabia; ^2^Department of Theriogenology, Faculty of Veterinary Medicine, Assiut University, Assiut-71526, Egypt; ^3^Department of Soil, College of Agriculture and Veterinary Medicine, Qassim University, Buraydah, Saudi Arabia; ^4^Department of Animal Production, College of Agriculture and Veterinary Medicine, Qassim University, Buraydah, Saudi Arabia

## Abstract

This study aimed to investigate the association of different reproductive disorders with the status of serum trace elements and steroid hormones in ewes and goats. This study included 131 barren and 11 fertile (control) ewes and 94 barren and 9 fertile (control) goats. Animals were examined gynecologically for reproductive soundness. The animals were bled, and their serum was harvested and assayed for manganese (Mn), selenium (Se), iron (Fe), zinc (Zn), estrogen (E2), and progesterone (P4) levels. The results showed that genital affections were associated with significant changes in serum Se (*P*=0.001), Fe (*P*=0.008), and Zn (*P*=0.01) levels in ewes, as well as Mn (*P*=0.01) levels in goats. Ewes and goats with cystic ovaries had higher serum Se, Fe, and Zn levels (*P*=0.0001) than ewes with uterine affections, ovarian inactivity, and controls. Ovarian inactivity was linked to low Se levels in ewes and low Se and Zn levels in goats (*P*=0.05). Ewes and goats with estrogen-dominant reproductive disorders had higher Se (*P*=0.01), Fe (*P*=0.03), and Zn (*P*=0.01) compared with the control group. Se (*P*=0.02) and Zn (*P*=0.05) were lower in ewes and goats affected with P4-dominant genital disorders compared with the control group. It can be concluded that the reproductive disorders are associated with changes in the level of trace elements in blood of ewes and goats. There is a reciprocal relationship between the levels of estrogen and progesterone with those of the trace elements in serum of ewes and goats.

## 1. Introduction

The role of minerals and trace elements in reproduction is unquestionable [1]. Trace elements are key players in the sexual activity of ruminants as part of the enzymatic build-up, hormonal secretion, antioxidant system, and immune function [2]. Reproductive failure may be brought by insufficiency of single or collective elements and by disproportions [3].

Manganese (Mn) is inevitable for fertility in ruminants. Number of services per conception, one of the fertility indices, was utterly more than the normal index in small ruminants raised on low Mn diets [4]. Low manganese resulted in a poor conception rate and increased embryonic mortality [5]. There are evidences suggesting that Mn might be essential for the activity of definite endocrine organs. The ovary and pituitary gland are comparatively rich in manganese [6].

Selenium (Se) is an antioxidant and a cornerstone in the enzymatic construction, especially glutathione-built ones. Deficiency is a main cause of premature birth, stillbirth, retained placenta, metritis, and mastitis in small ruminants [7]. Lambing has amplified when selenium was administered orally in monthly doses commenced one month before breeding and unending through pregnancy [3]. Administration of vitamin E and Se injections improved the pregnancy rate from 64.0 to 93.3% in sheep [8].

Iron (Fe) is greater in blood of cyclic than in noncyclic female sheep [9]. It is irreplaceable for hematogenesis. Correlations were strong between iron and zinc content of blood and the number of inseminations per conception [10].

Zinc (Zn) is indispensable in the production of many of the sex hormones, comprising steroid hormones and GnRH [11]. It is also elaborated in ribonuclease activity and gamete maturation during their synthesis and improves their quality [3]. Zn has been documented as a cofactor and component of definite enzymes [12]. Ewes on low zinc diets have irregularity of the estrus period, early embryonic mortality, low conception rate, and decreased litter size [4]. Reproductive performance of sheep has been enhanced when rams and ewes received zinc supplementation [13].

The impact of elements deficiency on reproduction has been studied in different livestock and their role has been clarified through their supplementation in ration [14]; however, their specific relation with certain reproductive disorders is still needing more investigation in small ruminants in tropical and subtropical areas. Moreover, the role of steroid hormones in determining the level of trace elements in the circulation is still equivocal and needs further investigation. This study, therefore, aimed to study the association of different genital affections with the level of trace elements and steroid hormones in ewes and does in Qassim region, central Saudi Arabia.

## 2. Materials and Methods

### 2.1. Animals

A total of 131 ewes and 94 goats in Qassim region, a central area of Saudi Arabia, were used in this study. Animals aged 1–6 years weighed 45–75 kg and had a body condition score of 3 on average (scale from 1 to 5, where 1 is skinny and 5 is obese [15, 16]). They fed maintenance ration for breeding females (1231.43 ± 39.07 g/head/day, about 2% of the bodyweight) and water ad libitum. Animals were admitted to the veterinary hospital for breeding soundness examination after being barren for 1–3 breeding seasons. Control groups of 11 and 9 of reproductively sound; fertile ewes and goats were randomly selected from the same cohort of the studied ewes.

### 2.2. Clinical Examination

The external genitalia were assessed by visual inspection. In order to monitor the reproductive tracts of the studied animals, transabdominal and transrectal ultrasonography was performed using a 5 MHz linear probe (Aloka Co., Ltd., Tokyo, Japan). Different genital affections were specified and recorded [17].

### 2.3. Blood Sampling

Blood was collected from the jugular vein of all admitted ewes and goats at 8:00–10.00 a.m. before the clinical examination in plain test tubes. For referencing to normal values of the assigned trace elements, control animals were bled in the context of routine herd health management and diagnostics. Samples were left at the room temperature to clot and centrifuged at 3000 ×g for 10 minutes. Serum was harvested and stored at −20°C until trace elements and hormonal estimation.

### 2.4. Estimation of Trace Elements in Serum

Serum was digested using HClO4-HNO3 mixture [18]. Concentrations of Mn, Se, Fe, and Zn were estimated by flame emission atomic absorption based on the previously described technique [19].

### 2.5. Hormonal Analysis

ELISA was used to estimate serum E2 and P4 using commercial kits (HUMAN Gesellschaft fur Biochemica und Diagnostica, Wiesbaden, Germany). The intraassays of the coefficients of variance were 3.7 and 5.1% and interassays 6.4 and 6.8% for E2 and P4, respectively. The sensitivity for E2 was 5 pg/mL and 0.15 ng/mL for P4.

### 2.6. Statistical Analysis

The data were presented in means ± SE. SPSS program, version 25 (SPSS Inc., Chicago, IL, USA, 2017), was used for statistical analysis. Differences between groups were analyzed by ANOVA. Relationships were estimated by the correlation coefficient. Significance was set at *P* < 0.05.

## 3. Results

The prevalence of different genital affections in barren ewes and goats is given in Tables 1 and 2, respectively. Endometritis was the most common genital affection in the studied animals.

Genital disorders were associated with significant changes in serum Se (*P*=0.001), Fe (*P*=0.008), and Zn (*P*=0.02) in ewes. In goats, genital affections were associated with significant changes in the serum level of all the estimated trace elements Mn (*P*=0.01), Se (*P*=0.002), Fe (*P*=0.0001), and Zn (*P*=0.001). Ewes and does affected with cystic ovaries had higher levels of serum trace elements than those affected with other reproductive disorders or control groups. Meanwhile, significantly lower serum Se and Zn levels in goats and lower serum Se only in ewes were found associated with ovarian inactivity.

Significantly higher E2 levels (*P*=0.01) were found in ewes and does affected with cystic ovaries, while those affected with ovarian inactivity had lower levels (Table 3). Serum P4 was higher (*P*=0.03) in ewes and does affected with pyometra.

Se (*r* = 2.34, *P*=0.01), Fe (*r* = 3.21, *P*=0.03), and Zn (*r* = 3.76, *P*=0.01) correlated positively with E2, while Se (*r* = −2.53, *P*=0.02) and Zn (*r* = −1.73, *P*=0.05) negatively correlated with P4 in the studied ewes and does.

## 4. Discussion

Endometritis was the common cause of infertility in ewes and does in the present study. It is well documented that the uterus is more vulnerable to infection than any other part of the genital tract in small ruminants [20]. Infection during copulation, delivery, puerperium, and unsanitary clinical examination may jeopardize the uterine integrity [21]. The negative impact of P4 on Se and Zn may be of diagnostic importance in animals affected with uterine disorders in this study. As a component of the enzymatic system, especially glutathione peroxidases, Se is a corner stone in the oxidant: antioxidant balance [4, 22]. Over production of free radicals and reactive oxygen is seen by many as a major cause of cell death of the epithelial lining of the reproductive system of ruminants [23, 24]. The disruption of the uterine mucosa and consequent failure of maintaining pregnancy in repeat breeding sheep and goats has been blamed for a decrease of antioxidant activity in Se-deficient diets [5]. On the other hand, Zn is directly linked to the formation of arachidonic acid and consequently the secretion of prostaglandin F2*α* from the endometrium which responsible for the lysis of the corpus luteum, the main source of progesterone secretion in ewes and does [2]. As most uterine affections are associated with persistence of the corpus luteum, it can be speculated that Zn deficiency is predisposed for these disorders in the studied animals.

Earlier reports pointed to a decrease in the immune defense mechanism during progesterone-dominant reproductive stages, partially due to decrease in the vasculature of the reproductive system and partly due to the quiescence of the reproductive musculature [25]. In this context, it has been emphasized that Zn is important for maintaining the intimacy of the uterine mucosa and its involvement in the immune system in small ruminants [14]. Although insignificant, Zn was lower in ewes and does affected by uterine infections. This is may explain the association between the negative impact of low Zn and the prevalence of uterine affections in the studied animals. Zn is prevalent in blood, and it is required for the activity of abundant enzymes and transcription factors involved in cellular protein build-up [26]. It is seen by many as the centerpiece for regenerating the epithelial lining of the uterine mucosa [27]. It has been supposed [2] that a prospective role of zinc in oogenesis might be to preclude destruction of oocyte DNA by inhibiting the DNase activity. These contributions might be enabled by effects on pituitary gonadotropin hormones FSH and LH [14].

Serum trace elements were higher in ewes and does affected with cystic ovaries and lower in those with static ovaries. Selenium, Fe, Zn, and Mn are fundamental for determining follicle growth, maturation, and dominance. It combats the effect of free radicals and maintains the assembly of the normal follicle [12, 24]. The role of Se and Zn in the secretion of gonadotropin hormones is indispensable. However, their serum increase concomitant to overproduction of estrogen is a bad tiding for the process of ovulation and predisposes for cyst formation [2] which may explain the positive association between a rise in serum trace elements, estrogen, and the incidence of cystic ovaries in the present study. It has been found that high Se levels may predispose to abundant free radicals production toxic for cells in the gonads, including the oocyte [28]. The relationship between E2 and trace elements has been investigated previously [29]. Administration of extrinsic estrogen was associated with a rise in the level of trace elements in blood [14]. Changes in plasma minerals in rats parallel changes in plasma estrogen during the estrous cycle, after ovariectomy, and during administration of exogenous estrogen [3].

It can be concluded that the reproductive disorders are associated with alterations in the level of trace elements in serum of ewes and goats. Ewes and goats affected by ovarian inactivity have low serum Se and Zn. Ovarian cysts are characterized by a rise in serum trace elements in small ruminants. Therefore, trace elements are probably involved in the pathogenesis of some reproductive disorders in small ruminants. There is a reciprocal relationship between the levels of estrogen and progesterone with those of the trace elements in the serum of ewes and does.

## Figures and Tables

**Table 1:** Manganese (Mn), selenium (Se), ferrous (Fe), and zinc (Zn) serum levels in ewes with various reproductive disorders compared with control.

Reproductive status	*n*	Mn (mg/L)	Se (mg/L)	Fe (mg/L)	Zn (mg/L)
Control	11	2.99 ± 0.75^a^	0.62 ± 0.08^a^	7.91 ± 1.95^a^	9.15 ± 0.63^a^
Endometritis	98	2.39 ± 0.35^a^	0.37 ± 0.05^a^	5.01 ± 0.71^a^	3.31 ± 0.49^a^
Inactive ovaries	12	5.15 ± 0.63^a^	0.14 ± 0.09^c^	5.84 ± 1.14^a^	3.01 ± 0.18^a^
Hydrometra	7	4.18 ± 0.62^a^	0.35 ± 0.06^a^	6.37 ± 1.79^a^	4.35 ± 0.33^a^
Pyometra	8	5.86 ± 0.29^a^	0.89 ± 0.09^a^	9.64 ± 0.99^a^	9.11 ± 1.05^a^
Cystic ovaries	6	2.85 ± 0.69^a^	1.21 ± 0.37^b^	17.64 ± 4.21^b^	13.02 ± 1.04^b^

Values are in means ± standard error. Different superscript letters in columns are given for significantly different values (*P* < 0.05).

**Table 2:** Manganese (Mn), selenium (Se), ferrous (Fe), and zinc (Zn) serum levels in goats with various reproductive disorders compared with control.

Reproductive status	*n*	Mn (mg/L)	Se (mg/L)	Fe (mg/L)	Zn (mg/L)
Control	9	1.97 ± 0.79^a^	0.87 ± 0.02^a^	12.59 ± 3.06^a^	9.78 ± 0.49^a^
Endometritis	72	1.82 ± 0.55^a^	0.61 ± 0.04^a^	10.44 ± 1.58^a^	4.51 ± 0.05^a^
Inactive ovaries	12	0.54 ± 0.06^a^	0.21 ± 0.02^c^	10.18 ± 1.20^a^	2.01 ± 0.83^c^
Hydrometra	3	2.09 ± 0.11^a^	0.54 ± 0.09^a^	13.29 ± 2.16^a^	5.17 ± 0.12^a^
Pyometra	3	2.51 ± 0.18^a^	0.45 ± 0.06^a^	10.11 ± 1.16^a^	5.65 ± 0.12^a^
Cystic ovaries	4	5.13 ± 0.96^b^	1.89 ± 0.25^b^	22.52 ± 4.20^b^	12.17 ± 1.83^b^

Values are in means ± standard error. Different superscript letters in columns are given for significantly different values (*P* < 0.05).

**Table 3:** Serum estrogen (E2) and progesterone (P4) associated with genital disorders in ewes and does.

Reproductive status	*n*		P4 (ng/mL)		E2 (pg/mL)	
	Sheep	Goats	Sheep	Goats	Sheep	Goats
Endometritis	98	72	3.07 ± 0.24^a^	2.63 ± 0.60^a^	97.92 ± 6.32^b^	101.96 ± 11.50^a^
Inactive ovaries	12	12	1.49 ± 0.75^a^	1.61 ± 0.05^a^	72.85 ± 9.75^b^	77.01 ± 10.05^b^
Hydrometra	7	3	2.89 ± 0.50^a^	5.08 ± 1.33^b^	112.98 ± 13.17^b^	99.32 ± 7.01^a^
Pyometra	8	3	3.26 ± 0.86^b^	5.99 ± 1.21^b^	133.53 ± 12.81^a^	127.50 ± 7.01^a^
Cystic ovaries	6	4	1.88 ± 0.75^a^	1.01 ± 0.03^a^	156.86 ± 9.75^a^	277.24 ± 12.05^c^

Values are in means ± standard error. Different superscript letters in columns are given for significantly different values (*P* < 0.05).

## Data Availability

The data used to support the findings of this study are available from the corresponding author upon request.
